# The use of combination advanced therapies in psoriatic arthritis: results from a UK multi-centre audit

**DOI:** 10.1093/rheumatology/keaf037

**Published:** 2025-03-03

**Authors:** Hannah Jethwa, Tania Gudu, Laura C Coates, Philip S Helliwell, William Tillett, Abdalla Abuelmagd, Rajinder S Andev, Madiha Ashraf, Tanya Baqai, Ernest Choy, Dhivya Das, Nicola Gullick, Catherine Heighton, Alison Kinder, Ramasharan Laxminarayan, Gayatri Mittal, Penelope Morris, Sandeep Mukherjee, Anupama Nandagudi, Satyapal Rangaraj, Poonam Sharma, Hoda Temple, Beverly Traub, Vishal Paisal, Ryan Hum, Arani Vivekanantham, Robert Wilson, Deepak Jadon

**Affiliations:** Department of Medicine, University of Cambridge, Cambridge, UK; Rheumatology Department, Cambridge University Hospitals NHSFT, Cambridge, UK; Rheumatology Department, Cambridge University Hospitals NHSFT, Cambridge, UK; Nuffield Department of Orthopaedics, Rheumatology and Musculoskeletal Sciences, Oxford, UK; Leeds Institute of Rheumatic and Musculoskeletal Medicine, University of Leeds and Bradford Teaching Hospitals NHS Trust, Bradford, UK; Rheumatology Department, Royal National Hospital for Rheumatic Diseases NHS Foundation Trust, Bath, UK; Department of Life Sciences, University of Bath, Bath, UK; Rheumatology Department, Royal National Hospital for Rheumatic Diseases NHS Foundation Trust, Bath, UK; Nuffield Department of Orthopaedics, Rheumatology and Musculoskeletal Sciences, Oxford, UK; Nuffield Department of Orthopaedics, Rheumatology and Musculoskeletal Sciences, Oxford, UK; Rheumatology Department, Luton and Dunstable Hospital NHS Foundation Trust, Luton, UK; Cardiff Regional Experimental Arthritis Treatment and Evaluation Centre, Cardiff, UK; Cardiff Regional Experimental Arthritis Treatment and Evaluation Centre, Cardiff, UK; Rheumatology Department, University Hospitals Coventry and Warwickshire NHS Trust, Coventry, UK; University of Warwick Medical School, Coventry, UK; Rheumatology Department, University Hospitals of Leicester NHS Trust, Leicester, UK; Rheumatology Department, University Hospitals of Leicester NHS Trust, Leicester, UK; Rheumatology Department, University Hospitals of Derby and Burton NHS Foundation Trust, Burton on Trent, UK; Rheumatology Department, Royal National Orthopaedic Hospital NHS Trust, London, UK; Rheumatology Department, Countess of Chester Hospital NHS Foundation Trust, Chester, UK; Rheumatology Department, The Royal Bournemouth and Christchurch Hospitals NHS Foundation Trust, Bournemouth, UK; Rheumatology Department, Basildon and Thurrock University Hospitals NHS Foundation Trust, Basildon, UK; Rheumatology Department, Nottingham University Hospital NHS Trust, Nottingham, UK; Rheumatology Department, North West Anglia NHS Foundation Trust, West Anglia, UK; Rheumatology Department, Countess of Chester Hospital NHS Foundation Trust, Chester, UK; Rheumatology Department, University Hospitals of Derby and Burton NHS Foundation Trust, Burton on Trent, UK; Rheumatology Department, Nottingham University Hospital NHS Trust, Nottingham, UK; Rheumatology Department, Manchester University NHS Trust, Manchester, UK; Nuffield Department of Orthopaedics, Rheumatology and Musculoskeletal Sciences, Oxford, UK; Department of Life Sciences, University of Bath, Bath, UK; Department of Medicine, University of Cambridge, Cambridge, UK; Rheumatology Department, Cambridge University Hospitals NHSFT, Cambridge, UK

Rheumatology key message

- Combination advanced therapy is infrequently used for spondyloarthritis and psoriatic arthritis in UK rheumatology practice.

Dear Editor, The spondyloarthropathies are heterogeneous conditions involving different tissues and with related conditions that may demonstrate different cytokine profiles resulting in differential therapeutic efficacy and the need for combination therapy, possibly with two biologic drugs (combination advanced therapy; CAT) [1]. Anecdotally, CAT is used but there are few publications and no appropriately designed trials. Most reports are case series, though the use of a dual targeted biologic (ABT122 targets TNF and IL-17) in comparison with TNF alone has been reported [2]. In the latter study efficacy of ABT122 did not exceed TNF inhibition alone but there were no differences in adverse events. Haberman *et al.* reported the induction of remission in a patient with severe PsA using a combination of an IL12-23 inhibitor and a TNF inhibitor, again with no safety concerns [3]. In some cases, the combination of apremilast with conventional DMARDs and/or biologics has been reported, again with no increase in adverse events [4]. Finally, the emergence of other oral targeted synthetic DMARDs (tsDMARDs) has invoked the possibility of CAT with these agents and biologics: Shurey *et al.* reported a small case series where patients with PsA were treated with tofacitinib and a biologic (either IL12-23, IL23 or IL17 inhibitors) with good efficacy and no major safety concerns [5].

However, in a small case series from Leeds combination biologic therapy in PsA resulted in a high discontinuation rate due to adverse events. De Marco *et al.* reported a series of six patients with refractory psoriatic disease who had combined treatment with an anti-TNF-α and IL-12/23 agents [6]. Three of the six patients had their biologic treatment combination discontinued due to infection (severe skin infection, two chest infections and respiratory failure with recurrent chest infections); one patient had treatment discontinued due to development of chest sarcoidosis and the others had treatment discontinued due to non-response.

Of interest is the use of CAT in other disorders with the recent publication of the VEGA trial where better early outcomes in ulcerative colitis were obtained with a short induction regime of a combination of a TNF inhibitor and an IL-23 inhibitor, followed by IL-23 monotherapy, in comparison with either drug alone; adverse events were seen equally across the treatment groups although one case of tuberculosis was reported in the CAT group after the induction period [7].

In 2020 we conducted a multicentre survey of 16 UK-based rheumatology departments where physicians are involved in the British Psoriatic Arthritis Consortium (BritPACT) to gain a better understanding of efficacy, adverse events and rationale for the use of combined advanced therapies in clinical practice for patients with psoriatic arthritis and other cases of spondyloarthritis. The aim was to evaluate efficacy, any reported adverse events and rationale for use.

The use of CAT was restricted, and 13/16 centres did not have patients on CAT. Regarding patients with PsA, eight had been treated with CAT (Table 1). Of these eight, six patients were reported from one centre. Of the eight patients, one was on a combination of apremilast and omalizumab, the latter for severe asthma—this patient was also taking methotrexate and sulfasalazine and had an articular primary non-response to this regime. Of the other seven patients, six were on a combination of apremilast and a biologic, and one on a combination of tofacitinib and risankizumab. All patients had previously been treated with multiple biologic drugs. All seven patients had a good initial response to the combination, achieving either remission or low disease activity. Only one of the seven patients had discontinued the combination: for the others the mean duration of therapy at the time of the survey was 49 months. No serious adverse events or infections were reported, and six patients continued the combination.

**Table 1.** Summary of data from patients with PsA on CAT

Patient ID	Age/sex	Disease	Co-morbidities	Combination Rx	Duration on combo Rx	Prior bDMARDs/small molecule agents	Prior csDMARDs	Achieved LDA/remission Y/N	Well tolerated Y/N	Serious infections	Medication related AEs	Currently on combination therapy Y/N
1	60 F	PsA + PsO	HTN/OA	Apremilast (30 mg BD) + ustekinumab (90 mg 3 monthly)	24 months	ETN/ADA	MTX/SSZ	Y	Y	N	N	N—secondary failure
2	59 M	PsA + PsO	Obesity/MGUS/severe asthma	Apremilast (30 mg BD) + omalizumab (monthly) + MTX 20 mg weekly + SSZ 1 g BD	3 months	N/A	N/A	N	Y	N	N	N—primary failure
3	59 M	PsA + PsO	HTN/ALD cirrhosis/polycythaemia	Apremilast (30 mg BD) + ustekinumab (90 mg 3 monthly)	60 months	ETN/ADA/INFX	MTX	Y	Y	N	N	Y
4	30 F	PsA + PsO	Nil	Apremilast (30 mg OD) + secukinumab (300 mg monthly)	86 months	ETN/ADA/UST	MTX/CIC	Y	Y	N	GI intolerance on BD dosing	Y
5	42 F	PsA + PsO	Nil	Apremilast (30 mg BD) + secukinamb/ixekizumab/guselkumab	61 months	ETN/ADA/INFX/UST	MTX	Y—PsO moderate PsA	Y	N	N	Y
6	53 M	PsA + PsO	OA	Apremilast (30 mg BD) + ustekinumab (90 mg 3 monthly)	61 months	ETN/ADA/INFX/CZP/GOL	MTX/SSZ/LEF	Y	Y	N	N	Y
7	46 F	PsA + PsO	Nil	RIZANKIZUMAB (150 mg 3-monthly) + tofacitinib (5 mg BD)	11 months	ADA/SEC/IXE/GUS/APREM	MTX/CIC/LEF/SSZ	Y remission PsO; LDA PsA	Y	N	N	Y
8	43 M	PsA	Vitiligo	Apremilast (30 mg BD) + adalimumab (40 mg fortnightly) + MTX 25 mg weekly s.c.	14 months	INFX/ETN/GOL/UST/SEC/CZP/IXE/GUS	TOF	N	Y	N	N	Y

ADA: adalimumab; AE: adverse event; ALD: alcoholic liver disease; APREM: apremilast; BD: twice daily; bDMARD: biologic DMARD; CIC: ciclosporin; csDMARD: conventional synthetic DMARD; CZP: certolizumab pegol; ETN: etanercept; GI: gastrointestinal; GOL: golimumab; HTN: hypertension, INFX: infliximab; LDA: low disease activity; MGUS: monoclonal gammopathy of unknown significance; MTX: methotrexate; OA: osteoarthritis; SSZ: sulfasalazine; UST: ustekinumab; TOF: tofacitinib; IXE: ixekizumab; GUS: guselkumab; SEC: secukinumab; LEF: leflunomide; OD: once daily.

Two centres reported three patients with other spondyloarthropathies on CAT. One patient with ankylosing spondylitis and inflammatory bowel disease (IBD) was treated successfully with a combination of ustekinumab and certolizumab. One patient with enteropathic arthritis had a combination of ustekinumab (primary indication: IBD) and etanercept (25 mg weekly; primary indication: inflammatory arthritis) for a duration of 15 months. In this patient immunosuppression was temporarily held prior to incision and draining and antibiotic use for abscesses secondary to chronic hidradenitis supperativa: the abscesses were not felt to be exacerbated by CAT. Ustekinumab was not restarted due to lack of response of IBD and etanercept dose was increased to 50 mg for inflammatory arthritis. A third patient with enteropathic arthritis took a combination of adalimumab (primary indication: axial spondyloarthritis) and ustekinumab (primary indication: IBD). The duration of CAT was 12 months by the time of data collection and no adverse events had been noted.

This survey has revealed limited use of CAT in UK practice as regards PsA and spondyloarthritis, probably because of funding restrictions. In PsA the majority of the patients had failed at least two biologic therapies putting the patients into a ‘difficult to treat’ category, yet outcomes were generally good and treatment persistence high. In other spondyloarthropathies dual biologic therapy was used because of different target tissue involvement. As more biologics and tsDMARDs become available more trials are needed focusing on differential response and safety of combination advanced therapy.

## Data Availability

Data are available on request to the corresponding author.
